# Functional Movement Disorders in Children

**DOI:** 10.3389/fneur.2020.570151

**Published:** 2020-11-12

**Authors:** Anjali Chouksey, Sanjay Pandey

**Affiliations:** Department of Neurology, Govind Ballabh Pant Postgraduate Institute of Medical Education and Research, New Delhi, India

**Keywords:** movement disorders, children, tremor, dystonia, myoclonus

## Abstract

Functional movement disorders (FMDs) are not uncommon in children. The age at onset may have a bearing on the phenomenological pattern of abnormal movement, risk factors, and response to different treatment modalities in this age group. FMDs in children resemble their adult counterparts in terms of gender preponderance, but risk factors are quite different, and often influenced by cultural and demographic background. FMDs contribute to a significant proportion of acute pediatric movement disorder patients seen in emergency settings, ranging from 4.3 to 23% in different case series. The most common movement phenomenologies observed in pediatric FMDs patients are tremor, dystonia, gait disturbances, and functional tics. Various social, physical, and familial precipitating factors have been described. Common social risk factors include divorce of parents, sexual abuse, bullying at school, examination pressure, or other education-related issues, death of a close friend, relative, or family members. Physical trauma like minor head injury, immunization, tooth extraction, and tonsillectomy are also known to precipitate FMDs. The response to treatment appears to be better among pediatric patients. We aim to review FMDs in children to better understand the different aspects of their frequency, clinical features, precipitating factors, diagnosis, treatment, and outcome.

## Introduction

Functional movement disorders (FMDs) are part of the broad spectrum of functional neurological disorders (FNDs). Previously, FMDs represented a diagnosis of exclusion, but can now be identified in an inclusionary manner using phenomenological manifestations that are specific to them without reliance on the presence or absence of psychological stressors or suggestive historical clues (1). Different term that is interchangeably used for FNDs in the old literature includes hysteria, conversion disorder, psychogenic disorder, non-organic, and dissociative disorder. Although hysteria was first described in children by Ranvlin in 1748, it was continued to be believed as “not a disease of childhood, but one which is occasionally seen in early life” (2). But later in the 1850s, Briquet showed that it did occur in children, and as frequently as in adults (3). FMDs in children and adults share some common features but they are bound to have some differences as well because the age at onset has bearing on the risk factors, the phenomenology of abnormal movement, and response to different treatment modalities (4). The knowledge of these differences can help to improve our understanding of the pathogenesis of FMDs. Most of the existing literature on FMDs pertains to adults only, so there is a paucity of data in literature describing the epidemiology, phenomenology, risk factors, management, and prognosis of FMDs among children. In this review, we aimed to discuss these aspects of FMDs in children.

## Methods

We did a PubMed search on 31st May 2020 using different terms related to our review. A total of 12,054 articles on “functional movement disorder in children,” 3,535 articles on “pediatric functional movement disorders,” and 120 articles on “psychogenic movement disorders in children,” were retrieved. Using the term “Functional” has a practical problem as the majority of the searched articles are not relevant to the review and articles related to functional imaging, functional magnetic resonance imaging (MRI), and functional neuroanatomy are also added. After the removal of duplicates, non-English articles, and animal studies, 34 articles were selected for the final reference list, which was based on the relevance to the topic of review.

## Epidemiology

The frequency of FMDs among children evaluated in movement disorder clinics ranges between 2 and 3% in different studies (4–6). In a study done by Kozlowska et al., FMDs contributed to 17% of the conversion disorder cases in children (7). Most of the studies showed a higher prevalence of FMDs among girls as compared to boys with a ratio of 3–4:1 which is similar to the adult data (4, 6). The exact mechanism of this sex predilection of FMDs is still not known because of the complex interaction of social, cultural, and biological factors (8, 9). FMDs in children tend to have abrupt onset as compared to adults and account for 4–23% of the acute movement disorders (5, 10, 11). In such cases, history or psychopathology is often absent or misleading, as the emergency room is not an ideal setting for eliciting history related to the psychosocial stressor and a thorough evaluation with standardized questionnaires is also sometimes not feasible (12). However, a careful clinical examination can help in making the early diagnosis of FMDs in children.

## Phenomenology

FMDs can adopt the phenomenology of any known movement disorder seen with an organic cause (Table 1). The three most common observed phenomenology among pediatric FMDs patients included tremor, dystonia, and myoclonus (13–15). Functional gait is also not uncommon in children. Dale et al. reported in their study that 12 out of 52 children with acute movement disorder had FMDs including tremor (*n* = 10), myoclonus (*n* = 5), dystonia (*n* = 4), and tics (*n* = 1). Among these, 10 children also had associated gait abnormality (5). In our study of 25 children with FMDs, we observed different phenomenologies including tremor (*n* = 11; 44%), dystonia (*n* = 4: 16%), gait abnormality (*n* = 4; 16%), psychogenic tics (*n* = 6; 12%), writer's cramp (*n* = 2; 8%), myoclonus (*n* = 2; 8%), and abdominal dyskinesias (*n* = 1, 4%) (15). Ahmed et al. reported 11 children with FMDs with tics (*n* = 6), tremor (*n* = 4), and clonus (*n* = 1) (16). In a recent review published by Harris, the movements observed were tremor (32.4%), dystonia (29.5%), myoclonus (24.3%), gait disturbances (9.8%), and others (3.9%) which included tics, chorea, or psychogenic tetany (17). Other rare FMDs that are reported in children include speech disorder, bizarre limb movements, palatal tremor, convergence spasm, apraxia of eyelid opening, athetosis, and drophead (4, 17). The diagnosis of FMDs in children is made based on several factors such as the presence of precipitating factors, abrupt onset and social stressors on history, paroxysmal symptoms, clinical characteristics of distractibility, and variability, inconsistency, and incongruous with the organic movement. The different features that can help to differentiate FMDs in children from their organic counterpart are almost similar to those observed in adults. However, there are a few important differences between adult and pediatric FMDs in terms of phenomenology (Table 1) (13–15, 18).

**Table 1:** Clinical clues for different functional movement disorders in children.

**Functional movement disorders**	**Clinical characteristics**
Functional tremor	Entrainment Distractibility Co-activation or co-contraction sign Pause of tremor during contralateral ballistic movements Variability in tremor frequency, burst duration, axis, and/or topographical distribution Whack-a-mole sign^@^
Functional Dystonia	Rapid onset Fixed posturing at rest from the outset Variable resistance to passive manipulation Distractibility or absence of dystonia when unobserved Presence of pain Lack of sensory trick or overflow
Functional myoclonus	Variability in duration and/or distribution of jerks or of their latency (if stimulus sensitive) Fully suppressible Entrainable into rhythmic oscillations upon repetitive tapping tasks Predominance of axial or facial jerks
Functional gait	Fluctuation of impairment Excessive slowness of movements, hesitation “Tightrope” walking with exaggerated truncal sway while maintaining a narrow base Truncal instability with good targeting of nearby objects Continuous flexion of the toes Improvement with distraction Worsening with suggestion, Tripping propulsion with falls
Functional Tics	Not fully stereotypical Interference with speech or voluntary actions Lack of premonitory urge Inability to voluntarily suppress tics
Features common in functional movements disorders in children	The dominant side is more affected Functional dystonia may not be always fixed Myoclonus is relatively more common Distractibility, variability, suggestibility, and entrainment are easily demonstrable

@ *Emergence and worsening of tremor in a separate body part when an initially affected body part is suppressed by someone holding it down*.

## Pathophysiology

### Neurobiological Factor

Most of the data regarding the neurobiological basis of FMDs are based on studies done in adult patients. These functional studies support the link between emotional processing and FMDs by showing altered activation of brain areas involved in emotional processing and increased functional connectivity between emotional and motor areas (19). Despite physiologic evidence demonstrating that functional movement utilizes voluntary motor pathways, FMDs patients report a lack of voluntary control over their abnormal movements due to the impairment in self-agency. The sense of agency (i.e., the sense that one is controlling one's actions) is a process of retrospective assessment of the action and it has been localized to the temporo-parietal junction (TPJ), pre-frontal cortices and the cerebellum (20, 21).

### Emotional Processing

As compared to healthy controls, children with conversion disorders use two distinct strategies for emotion processing—(i) psychological inhibition of distressing emotions and memories in which children use extreme psychological inhibition to minimize subjective awareness of distressing self-relevant feelings and memories (ii) coercion-preoccupation in which children focus on and exaggerate negative affect in extreme ways and remains preoccupied with a specific loss or trauma. Interestingly, these patterns of emotional processing correlated with the type of conversion symptoms (22). For example, children who used psychological inhibition presented with movement disorder reflecting the failure of inhibition like tremors or tics. While children who used psychological coercion-preoccupation presented with symptoms reflecting exaggerated non-verbal signaling of distress and disability like bizarre swaying gaits, or the assumption of odd postures.

### Predisposing Factor

#### Genetic Factors

A family history of parental physical or mental illness has been also observed in some studies (5). Thus, the possible role of genetic factors and epigenetics has been hypothesized. However, the impact of gene–environment interactions in the pathogenesis of functional neurologic disorders is still not clear (23).

#### Childhood Abuse

It has been proposed that FMDs patients may be primed by several traumatic events during early childhood such as emotional, physical, and sexual abuse with subsequent precipitating factors triggering abnormal movements. Kozlowska et al. hypothesized that children with conversion disorders, including FMDs are likely to have a higher rate of unresolved loss and trauma, thoughts, feelings, and memories about specific life events. These past unresolved events serve as emotionally charged material that has the potential to precipitate FMDs under the influence of powerful emotional triggers (22). A recent study also demonstrated that childhood abuse burden is associated with left anterior insular volume reduction in FND patients (24). Thus it has been hypothesized that adverse life events may affect developmentally vulnerable neural circuits leading to aberrant neuroplastic changes which later facilitates increased predilection for FMDs. However, another recent study found that stressful life events and maltreatment are substantially more common in people with FND than in healthy controls and patient controls, but many cases report no stressors. Thus, not all FMDs patients have a history of stressors or trauma, and not all children who are exposed to these stressors develop FMDs (25).

#### Perfectionistic Personality Traits

It is also a risk factor for FMDs in children. Ferrara et al. observed the features of perfectionistic personality associated with high academic and extracurricular achievement in 37% of their FMDs patients and all of them were girls (4). Different dimensions of perfectionism include excess concern over mistakes, personal standards, parental expectations, parental criticism, and doubts about actions and organization (26). It has been proposed that when a child with such personality traits make mistakes that are not accepted in their environment or when they feel incompetent to handle any demanding situation, a cognitive scheme might be activated, favoring distorted judgments of reality, leading to self-perceptions of worthlessness and incompetence, irritability and therefore increasing the thinking problems as well as the corresponding physiological symptoms (27).

#### Co-existing Psychiatric Illness

Psychiatric illness may coexist with FMDs in pediatric patients, but it appears to be less common as compared to adult patients. Previous studies have reported psychiatry comorbidity in around 10% of pediatric FMDs cases (28). However, two recent studies reported around 50% psychiatric comorbidity rate in pediatric FMDs patients (4, 14). Older studies had a lower rate as the standardized psychiatric assessment was not performed in all such cases and, therefore, the rate of underlying psychiatric diagnosis may have been underreported. Children tend to have predominantly anxiety disorders and attention deficit hyperactive disorder in contrast to adults who predominantly have depression.

### Precipitating Factors

**Figure 1:**
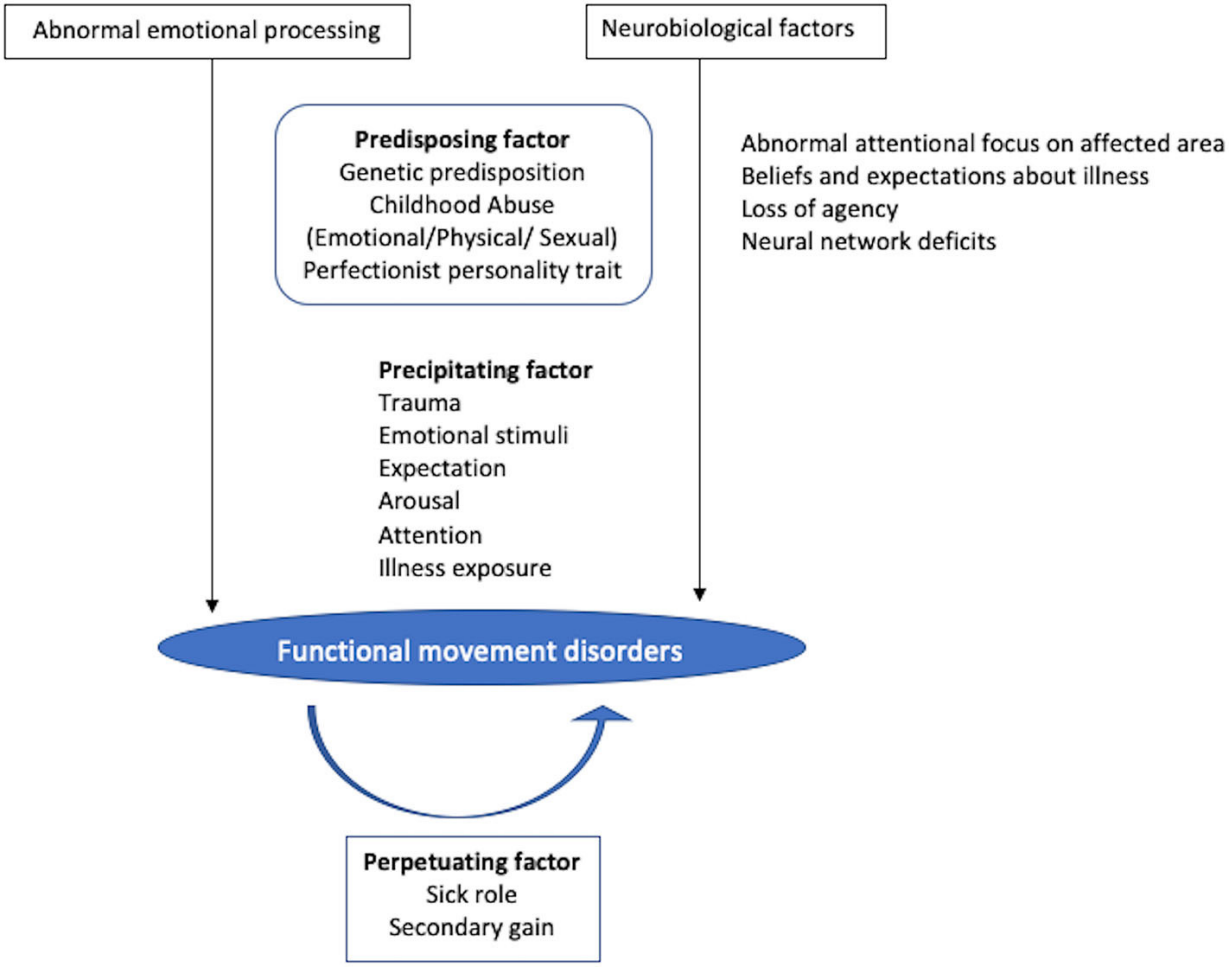
Pathogenesis of functional movement disorders in children. The two most critical mechanisms include abnormal emotional processing and neurobiological factors mainly impaired sense of agency, while various predisposing factors include genetic background, exposure to childhood abuse, and perfectionist personality trait. Different precipitating factors can activate neural mechanisms that modify normal sensory processing and thus override voluntary motor control triggering the abnormal movement. Once the functional movement disorders develop, the sick role and secondary gain act as a reinforcement factor. However, in many cases, specific predisposing or precipitating factors are not identified.

Various social, physical, and familial precipitating factors have been reported to trigger FMDs in children (Figure 1). These precipitating factors can activate neural mechanisms that modify normal sensory processing and thus override voluntary motor control. Common social factors associated with FMDs in the pediatric age groups include divorce of parents, death of a close friend, relative, or family member. Stressors at school like fear of examination or bullying at school are found to be particularly frequent in children with FMDs in many studies (5, 13, 15). This could be because of the great emphasis laid on academic performance by teachers and parents. Physical trauma like minor head injury, immunization, tooth extraction, and tonsillectomy has been reported to precipitate FMDs in the literature (5, 28, 29). Antecedent physical injury to the affected limb can precede the onset of functional dystonia in children. Parees et al. demonstrated the role of physical events preceding the onset of FMDs (30). Sometimes, children with FMDs have been exposed to others like a family member or a friend with illness. It can be explained by a phenomenon called “modeling,” defined as “the adoption of certain behaviors or motor patterns following the observation of close individuals displaying such manifestations.” Ferrara and Jankovic noticed the evidence of symptom modeling in around 11% of their pediatric FMDs patients (4). Another classical example of modeling includes “mass hysteria” rarely precipitating outbreaks of functional illness, particularly among adolescent girls (31).

### Reinforcing Factor

Once the FMDs develop, the sick role and secondary gain act as a reinforcement factor, particularly in children. The advantages of a sick role include staying home, abstaining from responsibilities, excessive pampering by parents, while the secondary gain acts through operant conditioning by providing a rewarding response following the symptoms. This include increased attention from family and friends, and relief of stress and pressure associated with the school (32).

## Differential Diagnosis

Clinicians often consider FMDs as a “diagnosis of exclusion,” not to be accepted until all other potential organic causes have been exhaustively ruled out, particularly in the pediatric age group. However, the recent DSM-5 criteria stressed on the demonstration of positive symptoms (33). Therefore, clinicians should be encouraged to make a phenotype-specific diagnosis. At the same time, it should not be made simply because of the presence of unusual symptoms, or the history of a stressor or prior psychiatric illness, or work up for the organic cause is normal. Few organic movement disorders of childhood can mimic FMDs as they may exhibit one or more of the above-mentioned features leading to misdiagnosis of FMDs, for example, paroxysmal dyskinesias, episodic ataxias, rapid-onset dystonia-parkinsonism, acute drug-induced dystonia, task-specific dystonia, dopa-responsive dystonia, and Tourette's syndrome. While some organic diseases can present with psychiatric manifestations first and movement disorders later such as Huntington's disease, Wilson's disease, and dentatorubropallidoluysian atrophy (34).

## Management

Various challenges faced while dealing with FMDs in childhood include—the lack of developmentally appropriate diagnostic interviews; the necessity to seek out and integrate multiple sources of information; and non-acceptance of diagnosis by parents, a higher rate of loss to follow-up; lack of training among clinicians for the recognition and management of FMDs in children.

While communicating the diagnosis of FMDs, terms such as “functional” or “psychogenic” should be avoided, instead, a detailed description of the illness should be provided. Two main factors are integral to the effective treatment of children and adolescents presenting with FMDs: (1) a multidisciplinary approach and (2) family involvement (35–37). A multidisciplinary approach involving neurology, psychiatry, and social work/psychology has been observed to be the most effective treatment strategy for children with FMDs. As a corollary, it is important to note that not all patients with FMDs require intervention for mental health. Patients with predominant motor symptoms may benefit from the guidance, self-help, and physical therapy program (38, 39). In such cases, premature psychiatric referral without adequate communication may further stigmatize their condition. Some adolescent patients and their families may feel psychological treatment to be unacceptable as they might initially be resistant to the idea that their symptoms are medically unexplained and essentially not organic; therefore, treatment within the medical setting may allow for increased engagement rather than referring the family to a mental health setting. Thus, it is ideal for assessment and treatment to occur within a hospital setting.

Involving parents and siblings in both the assessment and treatment plan of pediatric FMDs is important to ensure optimal effectiveness (40). During the assessment, family members provide invaluable information and insight into the patient's symptoms and the contribution of family dynamics to the patient's identified symptoms. Family members must understand that they affect the adolescent's symptoms. Lack of understanding of FMDs by parents and siblings might make them unsupportive and/or enabling of the child's condition. Enabling behaviors might result in the exacerbation of the “sick role” while their idea that the child is “faking” or pretending can also aggravate symptoms (41). Thus parents and other family members must learn the crucial role they play in the management of FMDs in children.

As mentioned above, stressors at school are common in children with FMDs. The school staff including teachers should also be included in the multidisciplinary team whenever possible. Even if the school is not actively involved, members of the multidisciplinary team should stay in touch with the school.

Recently, a greater role for physical therapy has been recognized in FMDs especially when motor symptoms predominate (42). During physical rehabilitation, the first step is to establish the treatment goal of relearning normal motor control and to avoid excessive attention to abnormal movements. Motor retraining begins by establishing elementary movements (e.g., weight-shifting) and consecutively adding more complex movements. Visual feedback during motor relearning from mirrors and videos can also be helpful.

Response to the placebo challenge is considered one of the diagnostic features of FMDs. Some studies showed a better response to placebo in children as compared with adults, which may be related to different perceptions of the illness, treatment, and underlying psychology (13, 43). However, placebo testing raises ethical concerns regarding a breach of the physician-patient relationship and is also not always curative.

## Prognosis

Early recognition and treatment of functional neurological symptom disorders can result in resolution or substantial improvement in 80–90% of childhood sufferers (15, 28, 44). However, the assessment of the remission rate of FMDs among children is challenging considering the high percentage of lost to follow-up because of non-acceptance of the diagnosis of FMDs by parents (4). In the study of Ani et al. including 204 cases of childhood conversion disorder, follow-up at 1 year was available for only 147 children. Out of them, around 90% showed an improvement in neurologic symptoms (45). Of the 52 cases of hysterical conversion reviewed by Grattan-Smith et al., 44% were symptom-free at discharge from the hospital and another 17% were markedly improved (46). In our study of 25 childhood FMDs patients, we noticed a complete improvement in 10 (40%), partial improvement in 9 (36%), and no improvement in 6 (24%) patients (15). Canavese et al. documented follow-up of 7 of 14 FMDs patients for a period ranging between 6 months and 4 years; of which 3 cases recovered fully, and 4 remained chronically disabled (14).

A brief duration of symptoms is reported as a good prognostic factor in the majority of the studies (28). When considering the phenomenology, children with tremor as predominant, FMD tend to have a more favorable prognosis. It might be because of the earlier diagnosis of FMDs in patients with tremor compared with other movement disorders (14, 28). Other factors like age at onset, comorbidity, stressors, or precipitants do not seem to affect the prognosis of FMDs in children (14). There is a lack of follow-up studies in the literature assessing the long term outcomes of FMDs in pediatric patients. Thus, it is not yet known whether the presence of childhood FMDs increases the risk of FMDs in adulthood.

## Conclusion

FMDs in children is a common disabling but potentially reversible condition that can be diagnosed clinically with a high level of certainty based on positive or inclusionary findings. Abnormal emotional processing and an impaired sense of agency are the two most critical mechanisms underlying FMD in children similar to adults. Different factors like genetic background, exposure to childhood abuse, and perfectionist personality traits may predispose an individual to develop FMD. However, in many cases, specific predisposing or precipitating factors are not identified. FMD in children differs from that in adults in terms of risk factors, phenomenology, and treatment response, and thus understanding of these differences has important clinical as well as research implication. Since the shorter disease duration carries an excellent prognosis in children, strenuous efforts should be made to reduce the time gap between symptom onset and diagnosis. The importance of appropriately communicating diagnosis to the patient and caregiver cannot be overemphasized which sometimes itself proves therapeutic. A multidisciplinary but individualized approach along with active participation by family members is the cornerstone of the successful management of FMDs in children.

## Author Contributions

AC contributed to data collection and manuscript preparation. SP contributed to manuscript preparation, review, and critique. All authors contributed to the article and approved the submitted version.

## Conflict of Interest

The authors declare that the research was conducted in the absence of any commercial or financial relationships that could be construed as a potential conflict of interest.
